# Immunotoxic effects of exposure to the antifouling copper(I) biocide on target and nontarget bivalve species: a comparative *in vitro* study between *Mytilus galloprovincialis* and *Ruditapes philippinarum*

**DOI:** 10.3389/fphys.2023.1230943

**Published:** 2023-08-15

**Authors:** Francesca Cima, Roberta Varello

**Affiliations:** Laboratory of Biology of Ascidians, Department of Biology (DiBio), University of Padova, Padova, Italy

**Keywords:** antifouling paints, bivalves, copper(I) chloride, hemocytes, immunotoxicity, mollusks, *Mytilus galloprovincialis*, *Ruditapes philippinarum*

## Abstract

Edible bivalves constitute an important bioresource from an economic point of view, and studies on their immune responses to environmental pollutants are crucial for both the preservation of biodiversity and economic reasons. The worldwide diffusion of copper(I)-based antifouling paints has increased copper leaching into coastal environments and its potential impact on both target and nontarget organisms. In this study, immunotoxicity assays were carried out with short-term (60 min) cultures of hemocytes from the bivalves *Mytilus galloprovincialis*—a mussel dominant in the macrofouling community—and *Ruditapes philippinarum*—a clam dominant in the soft-sediment community—exposed to CuCl to compare the toxic effects on their immune responses. The LC_50_ values were similar, 40 μM (3.94 mg L^-1^) for the mussel and 44 μM (4.33 mg L^-1^) for the clam. In both species, apoptosis occurred after exposure to 1 µM (98.9 μg L^-1^) CuCl, the concentration able to significantly increase the intracellular Ca^2+^ content. Biomarkers of cell morphology and motility revealed microfilament disruption, a significant decrease in yeast phagocytosis and lysosome hydrolase (β-glucuronidase) inhibition beginning from 0.5 µM (49.5 μg L^-1^) CuCl in both the mussel and clam. The same concentration of CuCl affected biomarkers of oxidative stress, as a significant decrease in reduced glutathione content in the cytoplasm and inhibition of mitochondrial cytochrome-c oxidase (COX) were detected in both species. Comparison of the biomarkers showed that clam is more sensitive than the mussel regarding alterations to the lysosomal membrane and reactive oxygen species (ROS) production, which supports the potential harmful effects of antifouling biocides on the survival of nontarget pivotal species in the coastal community.

## 1 Introduction

Bivalves are among the most specialized mollusks. This is due to the adaptive response to the benthic environment, in which they live and which generally consists of sandy-muddy bottoms or rocks. Bivalves living inside soft sediments such as clams are infaunal organisms that move and burrow using the hydrostatic pressure of their foot, while mussels are sessile forms of the epibenthos and live fixed to a hard substratum with the filaments of byssus secreted by a gland. For the different lifestyle, clams and mussels may come into contact with different sources of pollutants. Some edible species are considered good bioindicators because, as an important bioresource for human consumption, they are cosmopolitan and have been demonstrated to be very sensitive to various classes of pollutants. They are sessile (mussels and oysters) or mobile (clams), have a high filtration rate, may accumulate a wide range of contaminants in their tissues, play an important ecological role in the trophic chain of coastal environments and are a widespread commercial product (Goldberg, 1975). In recent decades, various species of bivalves have been tested in both field and laboratory studies as indicators of the water-sediment interface (Goulletquer and Heral, 1997; Blaise et al., 2002; Bebianno et al., 2004; Matozzo and Gagné, 2016). In particular, mussels of the genus *Mytilus* have been successfully tested worldwide in a number of field studies as sentinel organisms to examine the effects of pollutants in marine coastal waters (Goldberg, 1975; Kraak et al., 1992; Da Ros et al., 2000; Broeg and Lehtonen, 2006; Zorita et al., 2007; Cravo et al., 2009; Parisi et al., 2022). Functional responses have been used in the field of ecotoxicology as biomarkers or stress indexes to assess the effects of pollutants on environmental quality (Anderson, 1988). *In vitro* experiments are well suited to quickly screen various classes of pollutants and provide a significant amount of information about their mechanisms of toxicity (Carballal et al., 1997a; Carballal et al., 1997b; Pipe et al., 1997; Wootton et al., 2003; Cao et al., 2007; Donaghy et al., 2009; De Vico and Carella, 2012; Matozzo et al., 2012; Mosca et al., 2013; Messina et al., 2014; Pagano et al., 2016; Jiang et al., 2017).

The most diffuse coastal pollutants are chemicals used as biocides in antifouling paints. They are widely used to prevent fouling organisms such as serpulids, barnacles and mussels from settling to ship hull surfaces. Among all the different solutions proposed over the years, coatings with tributyltin (TBT) have been the most effective (Yebra et al., 2004). However, since the 1980s, many environmental problems have been reported in oyster farming in France with a significant decline in production. Shell thinning and malformations in oysters were correlated with TBT diffusion in harbors and marinas. Moreover, TBT has also proven an endocrine-disrupting agent causing imposex in mollusk species. It represents the acquisition of male sex organs by females leading to reproductive failure and a marked population decline (Bryan et al., 1986; Gibbs and Bryan, 1986; Coelho et al., 2006; Bigatti et al., 2009; Gibbs, 2009; Strand et al., 2009). In addition, TBT turned out to be extremely toxic to a number of aquatic organisms, particularly to those in sensitive early life stages (Thain and Waldock, 1986; Fent, 1996; Lignot et al., 1998).

Since 2001, when organotin compounds were banned worldwide by the International Maritime Organization, copper-based release technologies have been developed by the chemical marine paint industry and have progressively become the most widely used replacements for TBT-based coatings (Trentin et al., 2001; Voulvoulis et al., 2002; Yebra et al., 2004; Willemsen, 2005; Jones and Bolam, 2007; Lagerström et al., 2020). Metallic copper and copper(I) compounds, including cuprous thiocyanate, and cuprous oxide, are the dominant active substances in currently used antifouling paints (Voulvoulis et al., 2002; Yebra and Weinell, 2009; Cao et al., 2011). Copper is an essential micronutrient used in various metabolic processes; hence, low concentrations of this element are needed, but it starts to be toxic when it accumulates in tissues and exceeds the tolerance capacity of organisms (Xie et al., 2005). Speciation studies have indicated that in coastal waters, most copper is bound to organic ligands (Voulvoulis et al., 1999), and it can form lipophilic complexes with organic compounds, e.g., dithiocarbamates, increasing their toxicity by means of synergistic effects (Bonnemain and Dive, 1990). Consequently, the copper present in surface waters is mostly bound to organic materials with a high molecular weight and tends to settle in sediments. Only a limited amount of the total dissolved copper is bioavailable in the water column and, therefore, harmful to aquatic organisms. Dissolved copper can persist in the water column as Cu(II) or Cu(I) ions, where the latter has higher biocidal activity but could be easily oxidized to Cu(II). Anoxic conditions in sediments favor the stability of the reduced form, representing another long-term source of Cu(I) (Thomas and Brooks, 2010). High concentrations of dissolved copper have been commonly found in shallow, near-coastal marine areas (Biggs and D’Anna, 2012). The concentration of copper ions in marine sediments can be up to three times higher than that in the water column (Brooks and Waldock, 2009).

In the present study, a comparative evaluation of potential toxic effects of Cu(I) ions was conducted on a target and a nontarget species. The Mediterranean mussel *Mytilus galloprovincialis* (Lamark, 1819) and the Manila clam *Ruditapes philippinarum* (Adams and Reeve, 1850) were chosen as well-known filter-feeding bioindicators because they are dominant species in the trophic chain of the coastal ecosystem and are edible mollusks. The consumption of organisms collected from contaminated sites represents a potential risk for human health. These species are also widely used for baseline experimental bioassays and differ in their behaviors and habitats in coastal ecosystems. *Mytilus galloprovincialis* is a sessile species of hard substrata. It is common in the intertidal zone, where is dominant in the macrofouling community, and is an ideal sentinel organism for the presence of pollutants in the water column. It accumulates copper in tissues (Pospelova et al., 2018) and is a target species of antifouling control systems (Kim et al., 2009). Negative effects from copper(I)-based paints on the settlement of this species together with those of serpulids, bryozoans and ascidians were previously observed on steel and wood panels immersed for 10 months (Cima and Varello, 2023). As Cu(II) ions, subtoxic copper concentrations demonstrated negative effects on enzyme activities, electron transport reactions, membrane permeabilities and cell division (Davenport and Redpath, 1984; Pipe et al., 1999) and on the reproductive system by acting with protamine-like proteins (Lettieri et al., 2019). *Ruditapes philippinarum* is a mobile bivalve that locomotes through sediments using an enlarged, muscular foot. It is a nontarget species of antifouling control systems because it burrows in sandy-muddy bottoms but it could come into contact with adsorbed pollutants in the sediment. It is native to Japan, Korea, and the Philippines, from which it has been successfully imported since 1983 for human consumption in the Lagoon of Venice (northern Adriatic Sea) (Donaghy et al., 2009). In this species, bioaccumulation of copper as Cu(II) ions causes decrease in growth rate (Santana et al., 2017), and exposure to this pollutant disturbs energy metabolism and osmotic regulation in the gill tissues (Zhang et al., 2011).

Although copper(I) is considered one of the least toxic biocides of antifouling paints, previous studies have shown its potential to cause various damages to marine organisms and compromise their immune responses, development and survival (Bao et al., 2011; Kiaune and Singhasemanon, 2011; Cima and Ballarin, 2012; Tornero and Hanke, 2016). For this reason, the comparative evaluation of the Cu(I) ions toxicity has been carried out *in vitro* with biomarkers of immunotoxicity considering some functional responses of hemocytes from cultures of *M. galloprovincialis* and *R. philippinarum* since the immune system plays a fundamental role in organism survival. Alterations in immunosurveillance have been reported for bivalve mollusks exposed to metals (Cheng and Sullivan, 1984; Chen, 1988; Pipe et al., 1999) and xenobiotics (Fries and Tripp, 1980; Alvarez and Friedl, 1992; Beckmann et al., 1992; Coles et al., 1994; Cima et al., 1998; Cima et al., 1999). Pollutants may alter the functional parameters (i.e., biomarkers) of mollusk hemocytes, such as viability (Alvarez and Friedl, 1992), phagocytosis (Fries and Tripp, 1980; Anderson, 1988; Cima et al.*,* 1998), aggregation (Auffret and Oubella, 1997), adherence (Chen and Bayne, 1995), lysosomal enzyme activity (Cima et al.*,* 1999), and lysosomal membrane stability (Lowe et al., 1995; Grundy et al., 1996).

## 2 Materials and methods

### 2.1 Animals and experimental plan

*M. galloprovincialis* and *R. philippinarum* were collected along the southern coast of the Lagoon of Venice (near Chioggia, Italy) and immediately transferred to the laboratory. Organisms acclimated for 1 week before exposure to Cu(I) ions were reared in aquariums filled with filtered seawater (FSW, 35 psu, pH 8.1), which was changed every other day. They were kept in thermostatic rooms at 19°C, and the water temperatures in the experimental tanks were maintained at constant values using electronic thermostats. The bivalves were fed with Microbe-Lift^®^/Phyto-Plus B (Ecological Laboratories, Inc., Cape Coral, FL, United States of America) and microalgae (*Isochrysis galbana*). The collected bivalves were carefully checked for shell damage (damaged animals were not used in the experiments), and epibionts (i.e., barnacles and algae) were removed from the mussel shells. The experiments were performed outside the period of sexual maturity for mussels (winter to late spring, Da Ros et al., 1985) and clams (late spring to summer, Meneghetti et al., 2004) to reduce the potential for additional stress related to spawning during the experiments and to favor the energy balance towards the growth rate rather than the reproductive rate.

### 2.2 Copper(I)

Copper(I) chloride (purified, >99%, code 22433–2) was purchased from Aldrich Chem. Co., Milwaukee, WI, United States of America, as a water-soluble form of copper(I). Various nominal concentrations were obtained from a freshly prepared 10 mM stock solution in FSW, i.e., 0.05, 0.1, 0.5, and 1 μM, corresponding to 4.9, 9.8, 49.5, and 98.9 μg L^-1^ (0.9 × 10^−5^, 1.8 × 10^−5^, 0.9 × 10^−4^, and 1.8 × 10^−4^% by weight of Cu). Working solutions were always freshly prepared by dissolving drop-by-drop stock solutions in FSW to avoid the formation of precipitates and appeared clear for the entire experimental time. Bathocuproine (2,9-dimethyl-4,7-diphenyl-1,10-phenanthroline sulfonate; 96% pure, code 140910, Aldrich) was used at a concentration of 1 µM as a specific chelator of Cu(I) ions (White et al., 2001) to distinguish between the effects of Cu(I) and Cu(II) ions in the medium.

### 2.3 Hemocyte collection

Hemolymph was collected from the anterior adductor muscle with a 1-mL plastic syringe in the presence of 0.38% Na-citrate in FSW, pH 7.5, as an anticlotting agent. Once collected, the hemolymph was placed into a 1.5-mL Eppendorf tube and immediately centrifuged at 780 × g for 10 min. The pellet was resuspended in FSW and temporarily stored on ice. Sixty microlitres of hemocyte suspension was placed in the centre of a culture chamber or on a Superfrost Plus slide (Thermo Scientific, Gerhard Menzel B.V. and Co. KG, Braunschweig, Germany) depending on the assay. Culture chambers were set up with a Teflon ring (15 mm internal diameter and 1 mm thick), glued to the centre of a siliconized glass slide, and then smeared with Vaseline to be covered with a coverslip (Cima, 2010). Hemocytes were left to adhere to the glass substratum (in the case of the culture chambers, the glass substratum was represented by the coverslip after keeping the chambers upside down) for 30 min at room temperature. Culture chambers were used only for the LC_50_, lysosome stability index, phagocytosis index and glutathione index assays. For each experiment, three pools of hemocytes from five to six individuals were used.

### 2.4 Trypan Blue exclusion test for LC_50_ evaluation

This assay (Strober, 2015) was employed to determine the median lethal concentration (LC_50_), which is the compound concentration that is lethal for 50% of the cultured cells exposed for the experimental time (60 min). After adhesion, hemocyte monolayers were incubated with CuCl at 0.1, 1, 10, 20, and 50 μM, corresponding to 9.89, 98.99, 989.97, 1979, and 4929 μg L^-1^, respectively. At the end of exposure, the contaminant was discharged by two washes with FSW, and the hemocytes were incubated for 5 min with 0.25% Trypan Blue in FSW. This vital dye is excluded from functional/vital hemocytes but is retained in the cytoplasm of any senescent/dead hemocyte with altered plasmalemma permeability (Gorman et al., 1997). Observations, counting and micrographs of the hemocytes were performed directly on the culture chambers with an Olympus CX31 light microscope (LM) equipped with a Lumenera Infinity 2 digital camera and Infinity Capture Application v. 5.0.0 software (Lumenera Co. 2002–2009, Ottawa, ON). The percentage of dead hemocytes was determined by counting the number of cells with blue-colored cytoplasm, i.e., those unable to exclude the dye, divided by the total number of cells × 100. For the LC_50_ evaluation, the probit method (SPSS 11.0, SPSS Corp., Chicago, IL, United States of America) was performed. Based on the results obtained, the concentrations used in the subsequent acute toxicity experiments were considered sublethal.

### 2.5 Cell function assays

#### 2.5.1 Cytoskeleton stability index (F-actin content)

After adhesion and exposure to various concentrations of the contaminant, hemocytes were fixed in a 4% paraformaldehyde solution containing 0.1% glutaraldehyde, 1.7% NaCl and 1% sucrose for 30 min at 4°C, washed for 10 min in phosphate-buffered saline (PBS, 1.37 M NaCl, 0.03 M KCl, 0.015 M KH_2_PO_4_, 0.065 M Na_2_HPO_4_, pH 7.4). The monolayers were washed with PBS for 10 min and then permeabilized with 0.1% Triton X-100 in PBS for 5 min at 4°C. Hemocytes were then washed with PBS for 5 min and incubated for 30 min at 37°C in 3.5 µL of Acti-stain™ 488 Phalloidin (Cytoskeleton, Inc.) in 500 µL of PBS in the dark. Phalloidin binds in a specific and stable way to F-actin (microfilaments) but not to G-actin (monomeric), providing evidence of microfilament distribution in the cytoplasm. Finally, after rinsing with PBS, the slides were mounted and observed under the LM equipped with an amplified fluorescence by transmitted excitation of radiation (AFTER) LED fluorescence module (Fraen Corp., Corsico, Milan, Italy), with excitation at 470 nm. The cytoskeleton stability index was established by evaluation of the percentage of hemocytes with fluorescent cytoplasm due to the presence of nondepolymerized microfilaments with respect to the total hemocytes.

#### 2.5.2 Phagocytosis index

After adhesion, hemocyte monolayers were exposed to a yeast suspension (*Saccharomyces cerevisiae*; yeast:hemocyte ratio of 10:1) in FSW containing the four nominal concentrations of the contaminant reported above. After incubation for 60 min at 25°C, the slides were washed four times with renewed FSW to remove the nonphagocytized yeast cells. The hemocyte monolayers were fixed with 1% glutaraldehyde in PBS plus 1% sucrose. After washing with PBS, the monolayers were stained with 10% Giemsa dye in distilled water for 10 min, observed with an LM after several washes with distilled water and mounted with Acquovitrex aqueous mounting medium (Carlo Erba, Milan). The percentage of hemocytes with engulfed yeast cells with respect to the total was counted in 10 optical fields at a magnification of 1000× (Cima et al., 1998).

#### 2.5.3 Lysosomal stability index

Lysosomal membrane stability was assessed following a modified method of Lowe and collaborators (1995). A stock solution of neutral red (NR) (0.4%) was prepared in FSW, and the working solution was obtained by diluting 10 µL of the stock solution with 5 mL of FSW. After exposure of the hemocytes, 60 µL of NR working solution was added to each chamber. After 10 min, living hemocytes were observed under LM at 1000×. The lysosomal stability index is expressed as the percentage of hemocytes showing dye loss from lysosomes into the cytosol, which appeared reddish-pink.

#### 2.5.4 Cytosolic Ca^2+^ index

Sustained increases in cytosolic Ca^2+^ were revealed as dark-blue precipitates by Von Kossa’s substitution method (Schneider, 2021). Glutaraldehyde-fixed monolayers were immersed in 5% silver nitrate and exposed to ultraviolet light for 15 min. Cells were then rinsed with distilled water, incubated for 2–4 min in a 5% aqueous sodium thiosulfate solution, washed with distilled water, mounted in Acquovitrex, and observed under the LM at 1000×. The cytosolic Ca^2+^ index is expressed as the percentage of hemocytes containing precipitates.

#### 2.5.5 Apoptotic index

Late apoptosis was detected with the TUNEL reaction, which labels fragmented DNA within the nuclei. After adhesion and exposure to various concentrations of the contaminant, hemocytes were fixed in a 4% paraformaldehyde solution containing 0.1% glutaraldehyde, 1.7% NaCl and 1% sucrose for 30 min at 4°C, washed for 10 min with PBS and incubated in methanol plus 5% H_2_O_2_ for 30 min at room temperature to block endogenous peroxidase. The monolayers were washed with PBS for 10 min and then permeabilized with 0.1% Triton X-100 in 0.1% sodium citrate for 2 min at 4°C. The monolayers were then washed with PBS two times for 2 min each time and incubated for 60 min at 37°C with the reagents of the *in situ* Cell Death Detection, POD kit (Sigma‒Aldrich/Roche), containing FITC-labeled dUTP nucleotides and deoxynucleotidyl transferase (TdT). By means of TdT, labeled nucleotides bind to broken DNA strands at the free 3′-OH ends, which are as numerous as the DNA fragmentation that has occurred in apoptotic nuclei. At the end of incubation, the monolayers were washed three times with PBS, and the fluorescence signal was visualized by incubating the hemocytes for 30 min at 37°C with sheep anti-FITC antibody Fab fragments conjugated with horseradish peroxidase (CONVERTER-POD) provided in the same kit. The monolayers were finally washed three times with PBS and incubated for 10 min at room temperature in the peroxidase substrate (5 mg of 3–3′-diaminobenzidine (DAB, Sigma) dissolved in 200 μL of dimethyl sulfoxide plus 5 μL of H_2_O_2_ in 10 mL of PBS). The percentage of hemocytes with brown-colored nuclei compared to the total was taken as the apoptosis index.

### 2.6 Cytoenzymatic assays to evaluate the enzymatic index

#### 2.6.1 β-Glucuronidase

This enzyme (EC no. 3.2.1.31) is well represented inside the lysosomes of bivalves (Cima et al., 1999). It is a type of glycosidase that catalyses the hydrolysis of β-D-glucuronic acid residues from the nonreducing ends of mucopolysaccharides. After adhesion and exposure to various concentrations of the contaminant, hemocytes were fixed in 0.1% glutaraldehyde and then washed with sodium acetate buffer (0.1 M, pH 5.5) for 10 min and then incubated for 2 h at 37°C in the following incubation mixture: 4 mg of naphthol AS-BI β-glucuronide (Sigma‒Aldrich) dissolved in 250 µL dimethylformamide (DMF), 400 µL of solution A (0.4 g new fuchsin, Fluka), 2 mL of 36% HCl, 8 mL of distilled water, 400 µL of solution B (4% NaNO_2_ in distilled water) and 20 mL of 0.1 M sodium acetate buffer, pH 5.2 (Hayashi et al., 1964). After incubation, the hemocytes were washed with sodium acetate buffer for 10 min and mounted. The enzymatic index is expressed as the percentage of hemocytes showing red-stained (positive) sites of enzyme activity.

#### 2.6.2 Cytochrome-c oxidase (COX)

Cytochrome-c oxidase (COX, EC no. 7.1.1.9) is an enzyme in the mitochondrial respiratory chain, and its activity, which is related to mitochondrial functionality, is required for adenosine-5′-triphosphate (ATP) production (Syed et al., 2016). After adhesion and exposure to various concentrations of the contaminant, hemocytes were fixed in 0.1% glutaraldehyde and then washed with 0.1 M sodium acetate buffer (pH 5.5) for 10 min and then incubated for 2 h at 37°C in the following incubation mixture: 9 mL of 0.1 M acetate buffer and 0.2% DAB in 1% MnCl_2_ containing 0.01% H_2_O_2_ (Novikoff and Goldfischer, 1969). The enzymatic index is expressed as the percentage of hemocytes showing brown-stained (positive) sites of enzyme activity.

### 2.7 Oxidative stress assays

#### 2.7.1 GSH index

After adhesion and exposure to the various concentrations of the contaminant, living hemocytes were washed with FSW and then incubated for 10 min at 37°C in a 40 μM chlorobimane (Sigma-Aldrich) solution in FSW, which was obtained from a 20 mM stock solution in 95% ethanol. Chlorobimane has a high affinity for reduced glutathione (GSH) (Cookson et al., 1998). After at least two washes with FSW to eliminate the background fluorescence of the fluorochrome not bound to GSH in the cytoplasm, hemocytes were immediately observed under a LM equipped with an AFTER LED fluorescence module with excitation at 365 nm (UV). In this assay, the positive hemocytes appeared with blue fluorescence in the dark field, and the GSH index was obtained by calculating the percentage of fluorescent cells compared to the total, the latter of which were counted in the bright field.

#### 2.7.2 Superoxide anion index

After exposure to the contaminant, glutaraldehyde-fixed cells were incubated in 1 mg mL^-1^ nitroblue tetrazolium (NBT; Sigma-Aldrich) in PBS for 2 h at 37°C and then washed with PBS according to the method of Kim et al. (2009). Superoxide anions lead to the reduction of NBT by cleaving the tetrazolium ring to formazan, which precipitates as blue granules. After further washing with distilled water, the slides were mounted with Acquovitrex (Carlo Erba) for observation with a LM. The superoxide anion index is defined as the percentage of hemocytes containing dark blue spots of precipitated formazan.

### 2.8 Data analysis

Each experiment was replicated three times (*n* = 3), and the results are expressed as the average ±SD. The percentages of positive cells were evaluated by counting the hemocytes (at least 200 cells for each monolayer) in 10 optic fields at a magnification of 1000× (0.21 mm view field diameter). Values were normalized to control in FSW (100%). Data from the treated and control hemocytes were compared using one-way ANOVA followed by Dunnett’s test for multiple comparisons with DSAASTAT v. 1.1 2011 (Onofri, 2007). Differences were considered statistically significant when *p* < 0.05.

## 3 Results and discussion

Bivalves have an important ecological role and are widespread in aquatic ecosystems and as commercial products worldwide (Goldberg, 1975). Hemolymph contains two main types of circulating cells, i.e., hyalinocytes and granulocytes (Carballal et al., 1997a; Cima et al., 2000), which play key roles in the fundamental functions of organisms. They are involved in shell repair, digestion, excretion, and the immune response. Toxic effects due to interactions with pollutants can potentially impact the survival of organisms (Matozzo et al., 2001). The immunotoxic effects induced by Cu(I) in bioindicator species of water quality that belong to biofouling (mussel) or not (clam) have been considered in short-term *in vitro* assays. The indexes of immunotoxicity used as biomarkers showed that Cu(I) exposure had various effects on functional hemocyte responses.

The LC_50_ values of CuCl were determined to be 40 μM (3.94 mg L^-1^) for *M. galloprovincialis* and 44 μM (4.33 mg L^-1^) for *R. philippinarum*. In uncontaminated areas, the background copper concentrations within estuarine and coastal seawater range from 0.5 to 3 μg L^-1^ (Brooks and Waldock, 2009). According to the risk assessment of the European Union, the Predicted No Effect Concentration (PNEC), that is, the expected concentration without effect on populations of marine organisms, is 5.2 μg L^-1^ (ECI, 2008). In harbors with little water exchange and a high level of nautical activity, the copper released into the seawater by antifouling paints causes a significant increase in concentration that can exceed 20 μg L^-1^ (Schiff et al., 2007). Along the coasts of the Mediterranean Sea, copper concentrations have been found in the industrialized and urbanized areas ranging between 0.6 and 6.74 mg L^-1^ (Morroni et al., 2019). Therefore, the concentrations used in the ecotoxicological assays here (4.9, 9.8, 49.5, and 98.9 μg L^-1^) are within the range of copper concentrations in polluted aquatic environments and can be considered sublethal because they are all far below the LC_50_ values.

### 3.1 Effects on cell morphology

Pollutants can act on cytoskeleton proteins that ensure cell motility based on shape changes, cytosol fluidity and pseudopodium formation. Actin microfilaments consist of F-actin that assembles from globular monomeric actin or G-actin according to a model of dynamic instability that requires energy such as ATP (Romet-Lemonne and Jégou, 2021). Even in bivalve hemocytes, microfilaments are responsible for the formation of the cellular projections involved in cell motility and phagocytosis processes, such as pseudopodia and lamellipodia (Olabarrieta et al., 2001).

**FIGURE 1:**
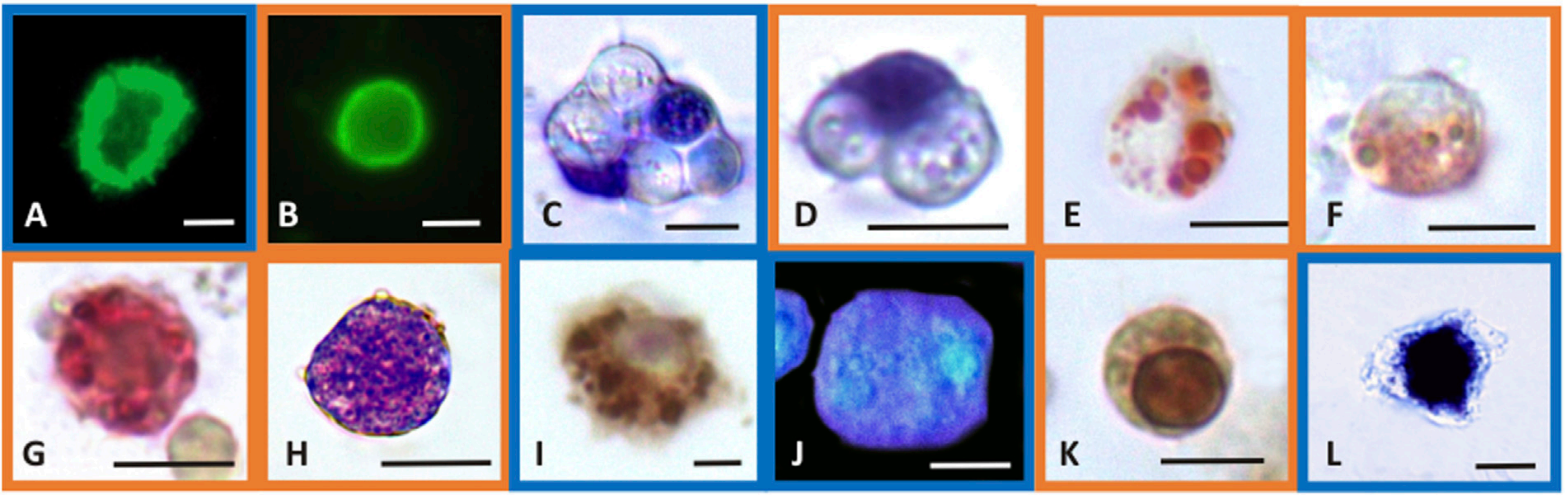
Selected light micrographs of *Mytilus galloprovincialis* (blue frame pictures) and R. philippinarum (orange frame pictures) hemocytes showing the histochemical features of the biomarker assays. Control hemocytes with F-actin distributed as stress fibers in the cortical layer of the cytoplasm, revealed by FITC-phalloidin in the assay of cytoskeleton stability **(A,B)**, and with ingested yeast cells in the phagocytosis assay **(C,D)**. In the latter, nuclei are blue stained with Giemsa dye. Lysosomal stability assay showing the neutral red dye retained inside the lysosomes of control hemocytes **(E)** and the release of the dye into the cytoplasm after exposure to CuCl **(F)**. Control hemocyte positive to the enzymatic assay for β-glucuronidase activity in the lysosomes that appear red-labeled **(G)**. Production of superoxide anions after exposure to CuCl revealed with the NBT assay as blue precipitates of formazan inside the cytosol **(H)**. Control hemocyte positive to the enzymatic assay for COX activity in the mitochondria that appear brown-labeled **(I)**. Blue fluorescence of reduced glutathione content in the cytoplasm of control hemocytes after treatment with chlorobimane in the GSH assay **(J)**. Hemocyte with nucleus stained brown by TUNEL reaction for the apoptosis assay indicating DNA fragmentation after exposure to CuCl **(K)**. Hemocyte with a large amount of calcium precipitates (black precipitates) inside the cytosol revealed with the Ca^2+^ content assay after exposure to CuCl **(L)**. Scale bars in pictures: 5 µm.

**FIGURE 2:**
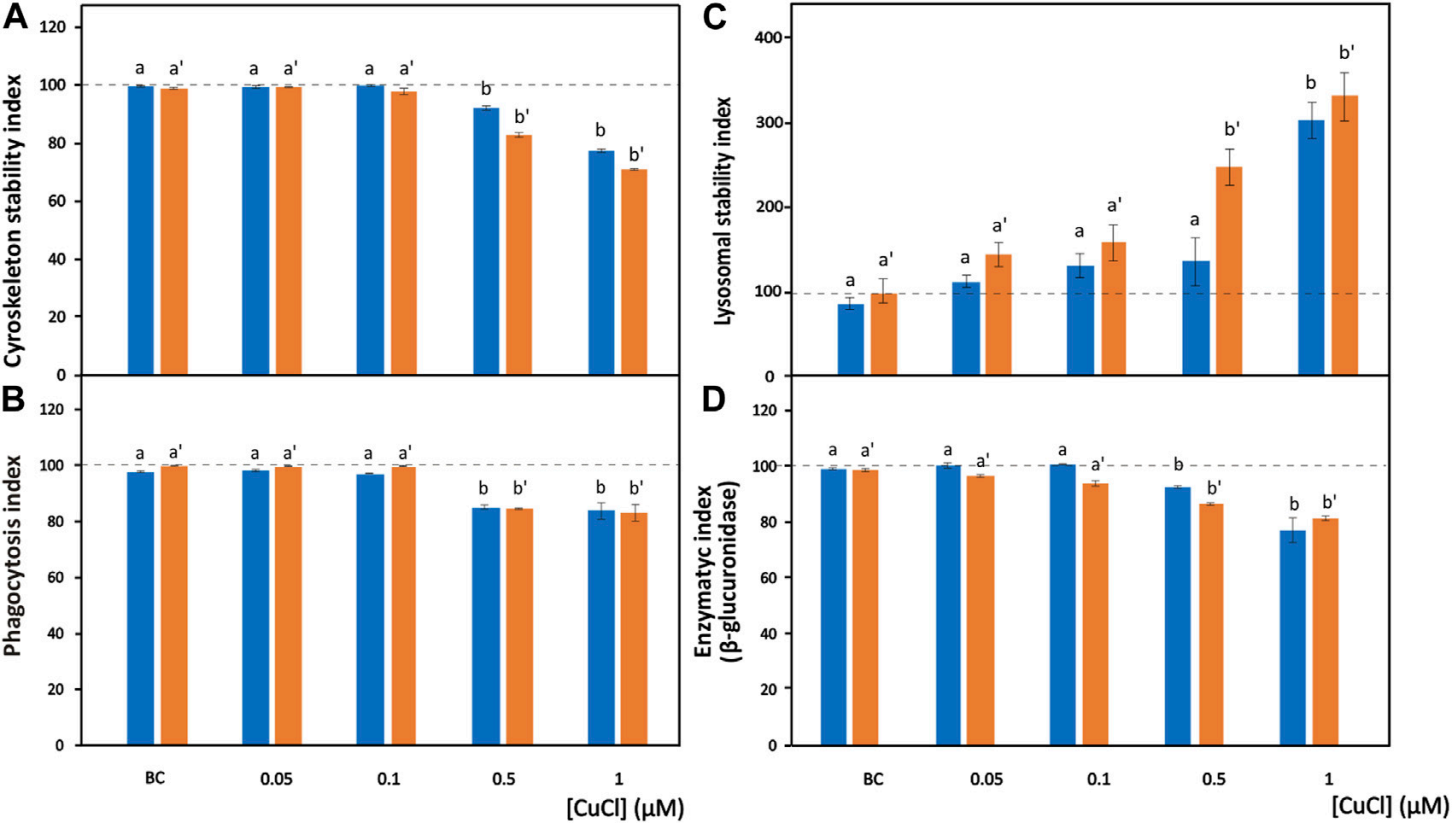
Effects of CuCl on the cell morphology and phagocytosis capability of *Mytilus galloprovincialis* (blue bars) and *Ruditapes philippinarum* (orange bars) hemocytes. Cytoskeleton stability indexes of F-actin content **(A)**, phagocytosis index **(B)**, lysosomal stability index **(C)** and enzymatic index of β-glucuronidase **(D)** in the presence of various concentrations of CuCl. Controls (100%, dotted line) in FSW. BC, the specific copper(I) chelator bathocuproine (1 µM) plus 1 µM CuCl. Data are shown as the mean ± s.d. *N* = 3. Different letters on histograms indicate significant (*p* < 0.05) differences for the pairwise comparison in *Mytilus galloprovincialis* (a to b) and *Ruditapes philippinarum* (a’ to b’).

Phalloidin is a toxin produced by *Amanita phalloides* that selectively binds to microfilaments, blocking their depolymerization and therefore stabilizing them (Dancker et al., 1975). Therefore, phalloidin can be used in laboratory assays when conjugated with fluorochromes to mark F-actin (Figures 1A,B). These assays highlight the disappearance of cytoskeletal microfilaments due to their disassembly provoked by toxic substances. After exposure to CuCl, a highly significant decrease (*p* < 0.001) in fluorescence beginning from a concentration of 0.5 μM (49.5 μg L^-1^) was found in both *M. galloprovincialis* and *R. philippinarum* (Figure 2A). Data on the effects of pollutants on the actin cytoskeletons of bivalves are generally scarce. The effects of exposure to Cu(II) ions have been well documented on only *M. galloprovincialis* hemocytes. Fagotti et al. (1996) first reported a cytoskeleton F-actin damage effect at concentrations of 5 μg L^-1^. Lethal and sublethal concentrations of Cu(II) ions from CuCl_2_ dissociation in water (3.17 × 10^5^ to 12.72 × 10^5^ μg mL^-1^) (Gómez-Mendikute and Cajaraville, 2003) and 0.02, 0.2, 1, 1.5 and 2 mg L^-1^ (Katsumiti et al., 2018), caused deleterious effects on F-actin. According to these authors, the harmful effects consist of the irregular organization of the cytoskeletal filaments of F-actin, which are poorly arranged and lead to decreased phagocytosis activity.

### 3.2 Effects on phagocytosis

In the cell-mediated innate immune responses of bivalves, phagocytosis is the main defense mechanism against pathogens and foreign materials (Cheng, 1981). This multi-step process involves recognition and adhesion of the target particle to the phagocyte plasma membrane, the ingestion of it in a heterophagic vacuole (phagosome), the fusion of lysosomes with phagosome containing the phagocytic material and the release of the lysosomal hydrolases contained within for intracellular digestion of the engulfed material (Cima et al., 2000).

The phagocytosis index describes the ability of hemocytes to phagocytize target particles such as yeast cells. These target cells are easily phagocytized by control (unexposed) hemocytes, which retain their original size but withdraw pseudopods to assume a spherical shape (Figures 1C,D).

In hemocyte cultures exposed to CuCl, the percentages of *M. galloprovincialis* and *R. philippinarum* cells that engulfed the target yeast cells significantly decreased beginning from a concentration of 0.5 μM (49.5 µg L^−1^) (*p* < 0.001) (Figure 2B). Similar results have been reported after bivalve hemocyte exposure to Cu(II) ions beginning from concentrations of 50 μg L^-1^ for *M. galloprovincialis* (Torres-Duarte et al., 2019) and 60 μg L^-1^ for *R. philippinarum* (Matozzo et al., 2001). These authors interpreted these observations by analyzing the relationship between the changes in phagocytic activity and alterations in cytoskeleton organization.

During phagocytosis, lysosomes have an important role because lysosomal hydrolases degrade foreign material engulfed inside heterophagic vacuoles. The hemocytes of bivalve mollusks may accumulate high levels of metals, mainly in the lysosomes (Pauley and Nakatini, 1968; Viarengo et al., 1981; Moore, 1990; Bordin et al., 1996). Alterations in the integrity of lysosomal membranes can lead to the release of such enzymes into the cytosol, resulting in damage to the cell itself (Lowe et al., 1995). For these reasons, the stability of lysosomal membranes can be evaluated as a useful biomarker of possible cellular damage through their capacity to retain the NR basic vital dye (Matozzo et al., 2001). NR is a cationic dye that can enter viable cells by pinocytosis or passive diffusion across the plasma membrane and accumulate in acidic intracellular compartments such as lysosomes (Coles et al., 1994). Healthy cells retain NR in their lysosomes that appear with a red-stained content (Figure 1E). Cells that exhibit lysosomal membrane alterations display an entirely red cytosol, since the dye diffuses outside the lysosomes (Figure 1F). Severe alterations in NR uptake have been reported in bivalve hemocytes of the genera *Mytilus*, *Dreissena* and *Ruditapes* collected from animals exposed to cadmium, lead and tributyltin (Coles et al., 1994; Gorman et al., 1997; Matozzo et al., 2001). These alterations to lysosomal membranes might be attributable to the high levels of metals that can accumulate in lysosomes, leading to the inactivation of Mg^2+^-ATPase, a proton pump that maintains the acid gradient inside the organelles. Dysfunction of this pump allows NR to pass from the inside of the lysosome to the cytosol. Alternatively, the accumulated metals can cause lipid peroxidation, leading to the destabilization of lysosomal membranes (Matozzo et al., 2001).

In the present study, a significant (*p* < 0.001) increase in the release of NR towards the cytoplasm from lysosomes was observed in the presence of 1 μM (98.9 μg L^-1^) and 0.5 μM (49.5 μg L^-1^) CuCl (Figure 2C) in *M. galloprovincialis* and in *R. philippinarum*, respectively. The results of previous studies utilizing this assay demonstrated a significant increase in toxicity by Cu(II) ions to *M. galloprovincialis* hemocytes, starting from a concentration of 1 mg L^-1^, which corresponds to a concentration of 7.43 µM (Katsumiti et al., 2018).

β-Glucuronidase is a lysosomal enzyme that hydrolyses carbohydrate polymers. It is widespread in bivalve mollusks, and its activity is used as a biomarker of pollutant toxicity (Cima et al., 1999). The cytoenzymatic assay (Hayashi et al., 1964) allows the *in situ* localisation of the activity of this enzyme (Figure 1G). β-Glucuronidase acts on the synthetic substrate naphthol AS-BI β-glucuronic acid by hydrolysing it. This reaction generates a product that binds with the diazonium salt pararosaniline, forming an insoluble red precipitate that makes the occurrence of the reaction identifiable within the cell (Cima, 2017). After exposure to CuCl, a significant (*p* < 0.001) decrease (Figure 2D) in the *in situ* enzyme activity was observed in the hemocytes of both species beginning from a concentration of 0.5 μM (49.5 μg L^-1^).

### 3.3 Oxidative stress

The actions of the lysosomal transport chain and lytic enzymes on the degradation of pollutants produces reactive chemical species (Galloway and Depledge, 2001), which are unstable and may have strong oxidizing actions on other cellular structures (Livingstone, 2001; Valavanidis et al., 2006). Reactive oxygen species (ROS) are molecules such as hydrogen peroxide (H_2_O_2_), the hydroxyl radical (^·^OH), the superoxide anion radical (O·^-^_2_) and the peroxyl radical (ROO^·^) that are generally produced during basal cell metabolism. However, a small percentage (2%–3%) of ROS can leave the compartments where these reactions occur and cause oxidative damage to various molecules essential for cell viability, such as phospholipids, proteins and nucleic acids. Therefore, cells develop a series of molecules and enzymes with antioxidant capacity that are able to react with ROS and prevent oxidative damage. Some pollutants may cause increased ROS production, exceeding the antioxidant capacities of the cell. The imbalance between the production and neutralization of ROS in the cell is called oxidative stress, and has become an important evaluation parameter in environmental toxicology (Kaloyianni et al., 2009). Consequently, changes in the activities of antioxidant molecules and enzymes are useful indexes in the evaluation of the toxicity of an environmental contaminant. In the hemolymph of *M. galloprovincialis*, extensive oxidative stress was indicated by various immunohistochemical parameters after exposure to metals like cadmium (Koutsogiannaki et al., 2014) and copper (Torres-Duarte et al., 2019) and a significant increase in the production of ROS, followed by oxidative damage identified by DNA fragmentation, protein carbonylation and lipid peroxidation, has been reported.

**FIGURE 3:**
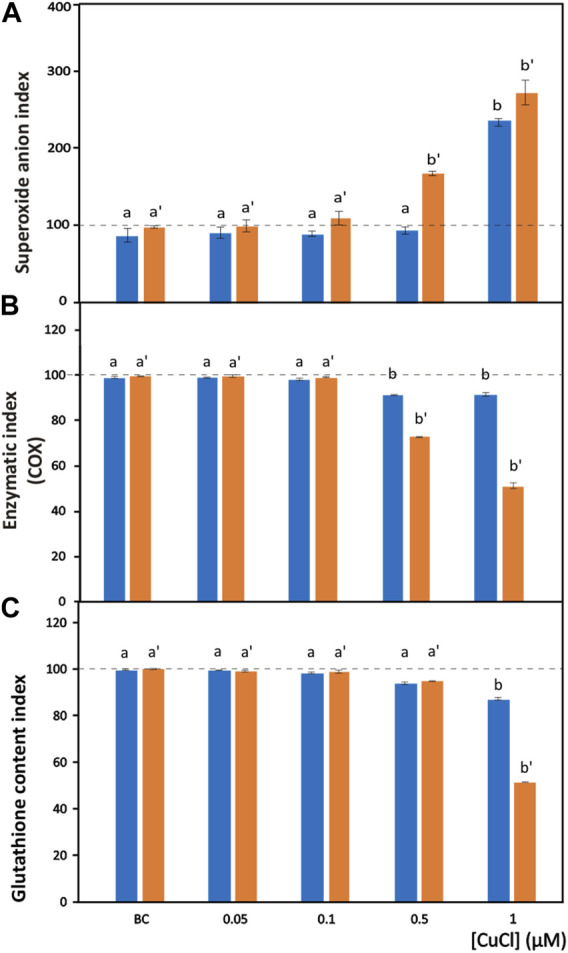
Oxidative stress induced by CuCl in the hemocytes of *Mytilus galloprovincialis* (blue bars) and *Ruditapes philippinarum* (orange bars). Superoxide anion index **(A)**, enzymatic index for COX **(B)** and GSH index **(C)** in the presence of various concentrations of CuCl. Controls (100%, dotted line) in FSW. BC, the specific copper(I) chelator bathocuproine (1 µM) plus 1 µM CuCl. Data are shown as the mean ± s.d. *N* = 3. Different letters on histograms indicate significant (*p* < 0.05) differences for the pairwise comparison in *Mytilus galloprovincialis* (a to b) and *Ruditapes philippinarum* (a’ to b’).

The superoxide anion radical is a main type of ROS produced in oxidative stress induced by pollutants and is the precursor of most ROS. Under normal conditions, the superoxide anion radical plays a positive role because it is a mediator in oxidative chain reactions (Bus and Gibson, 1982), as it can reduce cytochrome c in the intermembrane space of mitochondria (Wegerich et al., 2013). The mitochondrial matrix contains a specific form of superoxide dismutase (SOD), which then eliminates the formed superoxide anion radical (Turrens, 2003), but an excess of this radical due to pollutants can overpower its scavenging activity. Increased mitochondrial reactive oxygen species production, superoxide anions included, is responsible for the disruption and dysfunction of (COX), which is the terminal oxidase in the mitochondrial electron transport chain and is pivotal for ATP synthesis (Srinivasan and Avadhani, 2012). In the present paper, intracellular superoxide anion production revealed as formazan precipitates (Figure 1H) significantly (*p* < 0.001) increased after exposure to 1 μM (98.9 μg L^-1^) and 0.5 μM (49.5 μg L^-1^) CuCl (Figure 3A) in *M. galloprovincialis* and in *R. philippinarum*, respectively. The effects on ROS production by pollutants often show contradictory results depending on the exposure conditions used.

As an example, in previous experiments exposing *M. galloprovincialis* hemocytes to CuCl_2_ for 24 h, a significant decrease in intracellular superoxide anion production was reported (Gómez-Mendikute and Cajaraville, 2003). Most ROS are very reactive, have a very short lifetime and decompose very quickly even in the absence of external agents. In this scenario, the presence of low levels of superoxide anion radicals found might be considered in connection with a long exposure time to pollutants. Nevertheless, the progressive increase in oxidative damage is related to self-sustaining and amplifying actions triggered by ROS. A free radical can give up its unpaired electron to a nonradical, which, in turn, becomes a free radical capable of extending and propagating oxidative damage through a chain process. Therefore, the formation of a free radical often represents the initial, triggering event of the production of various molecules capable of damaging the normal redox state of the cell (Zorov et al., 2006).

The enzymatic index of COX was evaluated by determining the percentage of positive cells containing mitochondria that turned brown in the presence of the active enzyme (Figure 1I). After exposure to CuCl, this enzyme was significantly (*p* < 0.001) inhibited (Figure 3B) beginning from a concentration of 0.5 µM (49.5 μg L^-1^) in both species, indicating oxidative stress induced by the overproduction of ROS. This result is in agreement with previous observations on *R. philippinarum* hemocytes exposed to CuCl_2_, which revealed a decrease in enzymatic activity at the concentration of 110 μg L^-1^ (Matozzo et al., 2001). The mechanism of action of copper in mitochondrial metabolism and function is complex and only recently has been explained (Ruiz et al., 2021). At low concentrations, copper is essential for the assembly of COX and SOD inside mitochondria, which regulate a physiological ROS production promoting mitochondrial turnover and biogenesis. However, high levels of ROS like that provoked by Cu(II) complexes used in antitumor treatment in mammals are responsible of mitochondrial toxicity and DNA interactions, inhibiting cell proliferation and producing apoptosis.

Among molecules with antioxidant capacity, reduced glutathione (GSH) is a cytoplasmic tripeptide present in all animal cells. Its protective function against oxidative stress is due to the presence of a thiol group, which is reactive with ROS, decreasing their toxicity to the cell. By reducing other molecules, GSH is converted into its oxidized form, GSSG, which is able to react with electrophilic oxidants through a redox reaction. Thus, cell survival depends on the GSH/GSSG ratio. Glutathione can be considered an important biomarker that signals oxidative stress in the cell because the amount of GSSG depends on stressful conditions accompanied by the high production of ROS. When the cells are exposed to pollutants, the enzymes used to reduce GSSG to GSH, such as glutathione reductase and glutathione transferase are unable to convert the large amount of the oxidized form into the reduced form, and the cell undergoes oxidative stress (Liu et al., 2022). Chlorobimane allows the evaluation of GSH content in hemocytes, as the cytoplasm emits blue fluorescence when excited by UV light (Figure 1J). This assay showed a significant (*p* < 0.001) decrease (Figure 3C) in the number of fluorescent cells beginning from a concentration of 0.5 μM (49.5 μg L^-1^) CuCl in both *M. galloprovincialis* and *R. philippinarum*, suggesting that this contaminant can alter the oxidation state of cytoplasmic GSH with an increase in ROS production.

### 3.4 Induction of apoptosis

The phenomenon of apoptosis, or programmed cell death, is characterized by cell shrinkage and nuclear pyknosis and is accompanied by alterations in the integrity of the plasma membrane in the absence of inflammatory responses (Elmore, 2007). This irreversible phenomenon is considered to be related to both alterations in the homeostasis of cytosolic Ca^2+^ (Marinovich et al., 1996) and the induction of oxidative stress (Kannan and Jain, 2000).

**FIGURE 4:**
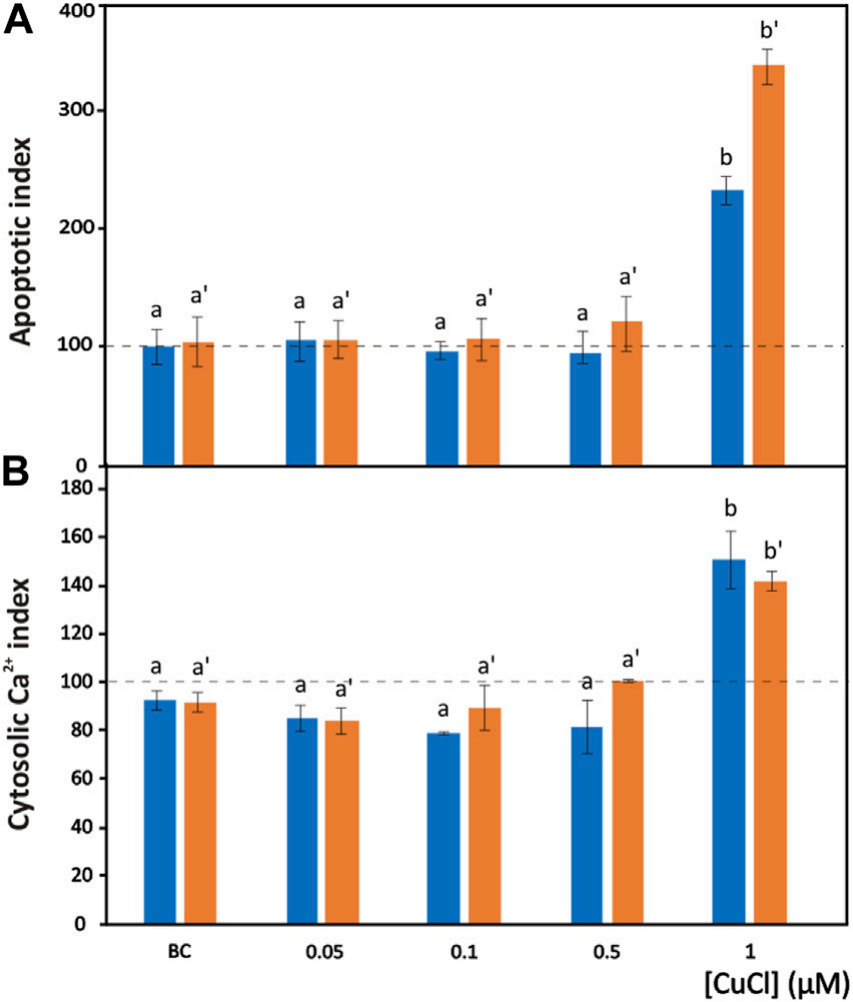
Apoptosis induced by CuCl in hemocytes of *Mytilus galloprovincialis* (blue bars) and *Ruditapes philippinarum* (orange bars). Apoptotic index **(A)** and cytosolic Ca^2+^ index **(B)** in the presence of various concentrations of CuCl. Controls (100%, dotted line) in FSW. Bc, the specific copper(I) chelator bathocuproine (1 µM) plus 1 µM CuCl. Data are shown as the mean ± s.d. *N* = 3. Different letters on histograms indicate significant (*p* < 0.05) differences for the pairwise comparison in *Mytilus galloprovincialis* (a to b) and *Ruditapes philippinarum* (a’ to b’).

The degree of chromatin fragmentation, i.e., the last event before cell blebbing, was evaluated by means of the TUNEL reaction (Figure 1K). Significant increases in the number of *M. galloprovincialis* and *R. philippinarum* cells with fragmented DNA were observed after exposure to 1 μM (98.9 μg L^-1^) CuCl (Figure 4A). At this concentration, the hemocytes became small, shrank, and underwent blebbing.

Evidence from several species of mollusks has supported the hypothesis that the induction of apoptosis in hemocytes is a cellular response to exposure to environmental pollutants such as herbicides, insecticides and drugs (Mitali et al., 2013; Ewere et al., 2021; Martyniuk et al., 2022). As an example, the gastropod *Lymnaea stagnalis* responds to exposure to various toxic substances by activating an apoptosis program involving lectins and increased ROS production (Russo and Madec, 2007). In particular, regarding the effects of Cu(II), a dose-dependent increase in apoptosis induced by oxidative stress has also been reported in the hemocytes of the bivalve *Perna canaliculus* after *in vitro* exposure (Nguyen et al., 2018).

At the same CuCl concentration able to induce apoptosis, a sustained increase in cytosolic calcium was observed in both *M. galloprovincialis* and *R. philippinarum* (Figure 1L; Figure 4B). This event consequently gives rise to a series of Ca^2+^-dependent toxic mechanisms in the cell, such as cytoskeleton disassembly, calmodulin inhibition, and endonuclease activation (Orrenius et al., 2003). These alterations could be caused directly by the binding of metal particles to proteins, followed by protein denaturation, or indirectly by a change in calcium metabolism due to oxidation of the thiol groups of cytoskeletal proteins by the increased presence of ROS. Viarengo et al. (1994) assumed that impairment of Ca^2+^ homeostasis observed in the hemocytes of *Mytilus edulis* is the consequence of a direct effect of the metal on Ca^2+^ channels. However, heavy metals, including copper, have affinities for the SH groups of proteins such as Ca^2+^-ATPase. Inactivation of this protein could lead to imbalances in calcium homeostasis and increased cytosolic Ca^2+^, causing severe alterations in the cytoskeleton and affecting important functions such as adhesion, motility and phagocytic capacity (Viarengo et al., 1993; Matozzo et al., 2001).

### 3.5 Comparative data from the literature

In the literature, data on the immunotoxic effects of copper to mussel and clam hemocytes have only considered Cu(II). A comparison of the toxic effects observed with Cu(I) in this study with previously reported results on the same specific biomarkers after bivalve hemolymph exposure to Cu(II) is summarized in Table 1.

**TABLE 1:** Comparison of toxic effects, evaluated *in vitro*, of Cu(II) and Cu(I) ions on clam and mussel hemocytes.

Biomarker	*R. philippinarum* Cu(II) ^1)^	*R. philippinarum* Cu(I)	*M. galloprovincialis* and *M. edulis* Cu(II)	*M. galloprovincialis* Cu(I) ^6)^
Cytoskeleton stability index (F-actin content)	-	49.5 μg L^-1^ ↓ (0.5 μM)	12.72 × 10^5^ μg mL^-1^ ↓ (9.46 × 10^5^ μM) ^2)^	49.5 μg L^-1^ ↓ (0.5 μM)
Phagocytosis index	10 μg L^-1^ ↓ (0.07 μM)	49.5 μg L^-1^ ↓ (0.5 μM)	50 μg L^-1^ ↓ (0.31 μM) ^3)^	49.5 μg L^-1^ ↓ (0.5 μM)
Lysosomal stability index	110 μg L^-1^ ↑ (0.81 μM)	49.5 μg L^-1^ ↑ (0.5 μM	1 mg L^-1^ ↑ (7.43 μM) ^4)^	98.9 μg L^-1^ ↑ (1 μM)
Cytosolic Ca^2+^ index	-	98.9 μg L^-1^ ↑ (1 μM)	135.8 μg L^-1^ ↑ (1 µM) ^5)^	98.9 μg L^-1^ ↑ (1 μM)
Apoptotic index	-	98.9 μg L^-1^ ↑ (1 μM)	-	98.9 μg L^-1^ ↑ (1 μM)
Enzymatic index (β-glucuronidase)	-	49.5 μg L^-1^ ↓ (0.5 μM)	-	49.5 μg L^-1^ ↓ (0.5 μM)
Enzymatic index (COX)	60 μg L^-1^ ↑ (0.45 μM)	49.5 μg L^-1^ ↓ (0.5 μM)	-	49.5 μg L^-1^ ↓ (0.5 μM)
GSH index	-	49.5 μg L^-1^ ↓ (0.5 μM)	-	49.5 μg L^-1^ ↓ (0.5 μM)
Superoxide anion index	60 μg L^-1^ ↓ (0.45 μM)	49.5 μg L^-1^ ↑ (0.5 μM)	-	98.9 μg L^-1^ ↑ (1 μM)

↑, increased and ↓, decreased effect relative to controls.

^1)^ Matozzo et al., 2001; ^2)^ Gómez-Mendikute and Cajaraville, 2003; ^3)^ Torres-Duarte et al., 2019; ^4)^ Katsumiti et al., 2018; ^5)^ Viarengo et al., 1994; ^6)^the present work.

The comparison of the responses after exposure to various Cu(I) concentrations of the two bivalves reveals that the immune system of *M. galloprovincialis* appears more resistant than that of *R. philippinarum.* In particular, the toxic effects of ROS production and lysosomal membrane permeability alterations were higher in *R. philippinarum* (0.5 µM compared to 1 µM of *M. galloprovincialis*). Concerning the differences in the mechanism of immunotoxicity at the subcellular level, Cu(I) generally shows significant detrimental effects on the cytoskeleton, stability of the lysosomal membranes, and increases in cytosolic Ca^2+^ at much lower concentrations than Cu(II). These different behaviors suggest the potential enhanced toxicity on molecular events by copper ions in the lowest oxidation state (Beswick et al., 1976; McBrien, 1980). The absence of toxic effects in the presence of bathocuproine, a Cu(I) chelator, confirms that toxicity is due to the presence of Cu(I) ions, and not Cu(II), in the medium, possibly due to its oxidation.

## 4 Conclusion

The endpoints investigated suggest that immunotoxicity is based on a potential mechanism of oxidative stress-immunotoxicity-cell toxicity of Cu(I) ions. This mechanism is multifaceted involving both direct and indirect interactions of copper. As other metal pollutants, copper can directly bind to thiol groups of various proteins in the cell such as cytoskeletal actin, membrane pumps, and enzymes causing their disassembly and/or inhibition. On the other hand, it can indirectly provoke a significant increase of ROS production from alteration of the electron transport chain in lysosomes and mitochondria with a consequent irreversible oxidative stress.

The direct interaction could explain the inhibitory effects observed on phagocytosis. Cytoskeleton disassembly and disorganization inhibit the formation of pseudopodia in phagocytes, which are fundamental for chemotaxis and the engulfing activity of non-self, potentially pathogenic cells and materials. By inactivating the lysosome pumps, copper causes damage on the membrane integrity and the consequent release, in the cytosol, of hydrolases, the activity of which is also inhibited by the pollutant. Consequently, phagocytosis is also negatively affected in both the formation of phagosomes and the intracellular digestion. In addition, the inhibition of the Ca^2+^-ATPase pump and the binding to calmodulin by Cu(I) ions are responsible of a sustained increase in cytosolic calcium, which causes a series of cell damages—also including indirect effects on cytoskeleton—leading to apoptosis.

The indirect interaction appears more complex. The inhibition of the electron transport chain triggers an unstoppable increase in ROS production such as superoxide anion, which cannot be countered by antioxidant systems (e.g., GSH) of the cell. Many adverse effects can be related to the excess of ROS activity such as protein denaturation, cross-linking, inhibition of enzymes, changes in the membrane permeability due to lipid peroxidation, and apoptosis induction.

Generally, mussels show higher resistance to copper than other marine invertebrates. In a previous comparative study on three species of marine invertebrates (*Carcinus maenas*, *Patella vulgata* and *M. edulis*), *M. edulis* was shown to be the most resistant species in a series of hemolymph assays that assessed differential sensitivity after exposure to CuCl_2_ for 7 days (Brown et al., 2004). This fact must be taken into consideration in toxicity assessment of copper-based antifouling biocides because high copper concentrations needed to limit the growth of fouling invertebrates could have serious repercussions on nontarget benthic invertebrates dominant in coastal ecosystems.

Both *M. galloprovincialis* and *R. philippinarum* have been proven to be good models to study immunotoxic effects of Cu(I) in organisms of biofouling and benthic infaunal habitats, respectively, because they can provide fundamental information on the mechanism of actions at both cellular and subcellular level of this pollutant released into the environment from antifouling paints. In addition, it must be borne in mind that high Cu(I) concentrations from various sources can potentially cause an increase in bioaccumulation with immunodepression and other underestimated harmful effects—e.g., reduced adaptability and survival capability, and reproductive failure—on nontarget pivotal species of bivalves of the coastal community living in the water-sediment interface such as clams. Further studies must be encouraged focusing on the dynamics of tissue bioaccumulation and acute-chronic toxicity of copper ions on nontarget bivalves because, as occurred in the past for other antifouling biocides, unexpected toxic effects might progressively deplete the clam population, endanger trophic chains and, no less important, have consequences on human health due to ROS-mediated oxidative stress from Cu(I) and Cu(II) biomagnification in the edible tissues.

## Data Availability

The original contributions presented in the study are included in the article/supplementary material, further inquiries can be directed to the corresponding author.
